# The epidemiology and biologics treatment patterns of juvenile idiopathic arthritis in Taiwan- an 8-year follow-up

**DOI:** 10.3389/fimmu.2025.1712103

**Published:** 2025-12-16

**Authors:** Ya-Chiao Hu, Yun-Lin Huang, Szu-Yu Yiao, Min-Ting Lin, Fang-Ju Lin, Yao-Hsu Yang

**Affiliations:** 1 Department of Pediatrics, National Taiwan University Hospital, Taipei, Taiwan; 2 Graduate Institute of Clinical Medicine, College of Medicine, National Taiwan University, Taipei, Taiwan; 3 School of Pharmacy, College of Medicine, National Taiwan University, Taipei, Taiwan; 4 Graduate Institute of Clinical Pharmacy, College of Medicine, National Taiwan University, Taipei, Taiwan; 5 Department of Medical affairs, Novartis (Taiwan) Co. Ltd., Taipei, Taiwan; 6 Department of Pharmacy, National Taiwan University Hospital, Taipei, Taiwan

**Keywords:** juvenile idiopathic arthritis (JIA), epidemiology, treatment, biologics, spondyloarthritis

## Abstract

**Background:**

Juvenile Idiopathic Arthritis (JIA) is the most common chronic arthritis in children, causing significant joint inflammation and complications. This study evaluates the incidence, prevalence, and treatment patterns of JIA in Taiwan, focusing on biologic therapy.

**Methods:**

A retrospective observational study utilized data from the National Health Insurance Research Database (NHIRD) for the period 2011-2020. Patients diagnosed with JIA were identified, and the annual incidence and prevalence rates were calculated. Treatment patterns, particularly regarding biologics, were assessed. Subgroup analysis compared patients diagnosed with spondyloarthritis (SpA) versus non-SpA within the JIA cohort.

**Results:**

Two thousand and thirty-three JIA patients were included. The annual prevalence ranged from 18.0 to 24.2 per 100,000 populations under 16 years old, with an incidence of 5.22 to 6.57 per 100,000. A male predominance was noted (male-to-female ratio: 1.08-1.52:1). The JIA-SpA subgroup comprised 49.2% of the JIA cohort. Compared to Non-SpA patients, the JIA-SpA subgroup showed male predominance (70.7% vs. 44.4%, p<0.0001), later age of onset, and lower biologic use (15.2% vs. 23.1%, p<0.001). Tumor necrosis factor alpha inhibitors were the most used biologics, with a continuation rate of 49.1%. Switching occurred in 13.2% of patients, mostly without interruption within a treatment gap of 60 days. Discontinuation without subsequent biologic therapy was observed in 9.5% of patients, while 28.2% restarted therapy after a treatment interruption of more than 60 days.

**Conclusion:**

This study highlights the epidemiological characteristics and treatment patterns for JIA in Taiwan, focusing on biologic therapies. Most patients maintained consistent biologics, with a small proportion able to discontinue them, emphasizing the need for region-specific management strategies.

## Background

Juvenile idiopathic arthritis (JIA), the most common cause of chronic arthritis in childhood, affects specific articular joints and results in arthritis and constitutional symptoms in adolescents or children under 16 years old. JIA is classified into seven subtypes based on the clinical and laboratory features identified in the first six months of illness (1, 2). The incidence and prevalence of JIA varied in different studies due to different genetic or environmental risk factors among populations. The prevalence rates range from 3.8 to 400 patients per 100,000 population, with an average estimate of 20.5 according to the systemic review (3). The prevalent rates differ widely among the seven JIA categories. According to the JIA studies in Europe and America, the most common subtype of JIA is oligoarthritis. Unlike these epidemiological data, the most prevalent JIA subtype in Taiwan is enthesitis-related arthritis (ERA) (4, 5), primarily diagnosed in male adolescents. Therefore, a region-specific cohort is needed to better understand JIA in Taiwan.

Pharmacological treatment for JIA aims to manage joint pain and inflammation, minimize joint damage, and prevent long-term complications such as loss of function and disability (6). The current treatments for JIA are based on American College of Rheumatology (ACR) guidelines, which involve a strategy of escalating treatment from non-steroidal anti-inflammatory drugs (NSAIDs) to conventional synthetic disease-modifying anti-rheumatic drugs (csDMARDs) according to the patients’ response to the treatment (7, 8). For patients with moderate to severe disease status, the biologics, mainly tumor necrosis factor alpha (TNFα) inhibitors are recommended as second-line therapy (7). Since the approval of etanercept for use in JIA in 1999, the use of biologics in JIA has increased. The trials of different biologics and accumulated experiences in the registry database (9, 10) demonstrated the efficacy of biologics in JIA disease control and minimization of joint damage. However, considering the long-term adverse effects and cost (11), the recommendation on whether and how to withdraw the biologics remained a challenge. A real-world experience is still lacking in understanding the treatment patterns of biologics in JIA.

This study aims to investigate the incidence and prevalence rates of JIA and explore the current treatment patterns in Taiwan, especially the use of biologics in JIA treatment. This study allows us to evaluate the differences between guideline-recommended treatments and treatments received in real clinical environments. The information will aid further clinical practice and health policies for the care of JIA in improving the clinical care of the affected population.

## Methods

### Study design and data

This retrospective observational study used data from Taiwan’s National Health Insurance Research Database (NHIRD) between 2011 and 2020. The NHIRD encompasses up to 99.99% of Taiwan’s population, representing approximately 23 million individuals, and provides a nationally representative cohort (12). The database includes comprehensive claims data such as patient demographics, visits, inpatient and outpatient diagnoses, procedures, and pharmacy prescriptions (13). For the inpatient and outpatient diagnosis codes, ICD-9-CM codes were used until the end of 2015, after which ICD-10-CM codes were used.

### Study population

The patient identification period was from January 1, 2012, to December 31, 2019, ensuring all participants met a minimum observation period to fulfill the observation criteria for at least one year. The date of the first diagnosis of JIA within the study period was defined as the Index date. The ICD codes used to identify JIA are listed in **Supplementary Table 1**. To define the prevalent JIA cohort, the following inclusion criteria were applied: (1) having at least one inpatient or three outpatient JIA diagnosis codes within 365 days between January 1, 2012, and December 31, 2019; (2) having enrollment record in Registry for Beneficiaries from 2012 to 2019; (3) under 16 years of age at the index date; and (4) having received systemic NSAIDs for more than 14 consecutive days within 365 days after the first JIA diagnosis. The incident JIA cohort included patients newly diagnosed with JIA between January 1, 2012, and December 31, 2019. The flowchart for enrollment for both prevalent and incident JIA cohorts is depicted in **Supplementary Figure S1**.

### Subgroup classification

To further characterize the JIA population in Taiwan, we identified a subgroup of patients with phenotypes consistent with spondylarthritis. Because the NHIRD lacks laboratory data (e.g., HLA-B27) and specific physical examination records required for strict ILAR classification of ERA (1), we adopted an operational definition termed JIA with Spondyloarthritis Features (JIA-SpA). Patients were classified into the JIA-SpA subgroup if they had concurrent diagnostic codes for ankylosing spondylitis, sacroiliitis, other inflammatory spondylopathies, or psoriatic arthropathy (detailed ICD-9 and ICD-10 codes are listed in **Supplementary Table 1**). Patients without these codes were classified as the non-SpA JIA group.

### JIA epidemiology

The prevalence and incidence of JIA were calculated from the data from 2012 to 2019. Annual prevalence rates were determined by dividing the total number of diagnosed JIA cases within a given year by the population of individuals under 16 years of age in that same year. Annual incidence proportions were calculated as the number of newly diagnosed JIA cases in a specific year divided by the population at risk (population under 16 years old without prior JIA diagnoses) during that year. Age- and sex-specific prevalence and incidence rates were also calculated based on the corresponding populations for each subgroup. Patient characteristics, including age, sex, month of diagnosis, geographic location, medical department, and hospital level, were analyzed to describe the enrolled JIA population.

### Treatment patterns and biologics survival

The pharmacologic treatments for JIA were listed based on the treatment guidelines of the American College of Rheumatology/Arthritis (7, 8). Standard therapies, including NSAIDs, systemic steroids, csDMARDs, and biologics, were analyzed (**Supplementary Table 2**). The grace period for determining drug discontinuation between prescriptions was set as 14 days for NSAIDs, systemic steroids, and csDMARDs, and 60 days for biologics.

The prescription patterns of three JIA biologics—etanercept, adalimumab, and tocilizumab—during the follow-up period were analyzed in the incident cohort. We omitted abatacept in the analysis due to the limited number of cases and its position as a second-line biologic option for JIA treatment. The first biologic prescribed to a patient was defined as the index biologic. Treatment continuation was defined as uninterrupted use of the index biologic without a gap exceeding 60 days. A treatment switch was defined as a transition from the index biologic to another JIA biologic. Treatment switching occurred within the 60-day treatment gap, while treatment switching after interruption was defined as a switch occurring after this period. As no standard definition of treatment gaps for biologics exists, the 60-day threshold was based on biologic maintenance dosing schedules and **clinical** tapering practices. Treatment interruption was defined as a period exceeding the 60-day gap without further drug supply. Discontinuation referred to the absence of biologic prescriptions after treatment interruption until the end of the follow-up. If a patient resumed biologic therapy following an interruption, this was classified as a treatment restart.

The treatment duration for each biologic was calculated exclusively for the index biologic. Drug survival was measured in consecutive days from the initiation of the index biologic until the earliest occurrence of discontinuation, treatment switching, or study end, whichever occurred first. Additionally, the concurrent use of csDMARDs as add-on therapies during biologic treatment was assessed by identifying csDMARD prescriptions issued within the same outpatient visit or included in the same inpatient order as the biologic prescription.

### Statistical analysis

In descriptive data analyses, categorical variables were presented as frequency counts and percentages, and continuous variables were presented as means (standard deviation [SD]) or medians (interquartile range [IQR]). Comparisons of categorical variables were conducted using the chi-squared test or Fisher’s exact test. For treatment patterns, metrics such as drug survival, time to switch, and time to restart biologics were estimated in the incident cohort using the Kaplan-Meier method. In accordance with NHIRD regulations, all non-zero counts below three were suppressed to protect patient privacy.

All statistical analyses were performed using SAS 9.4 (SAS Institute Inc, Cary, NC, USA).

## Results

### Epidemiology and clinical characteristics

**Figure 1:**
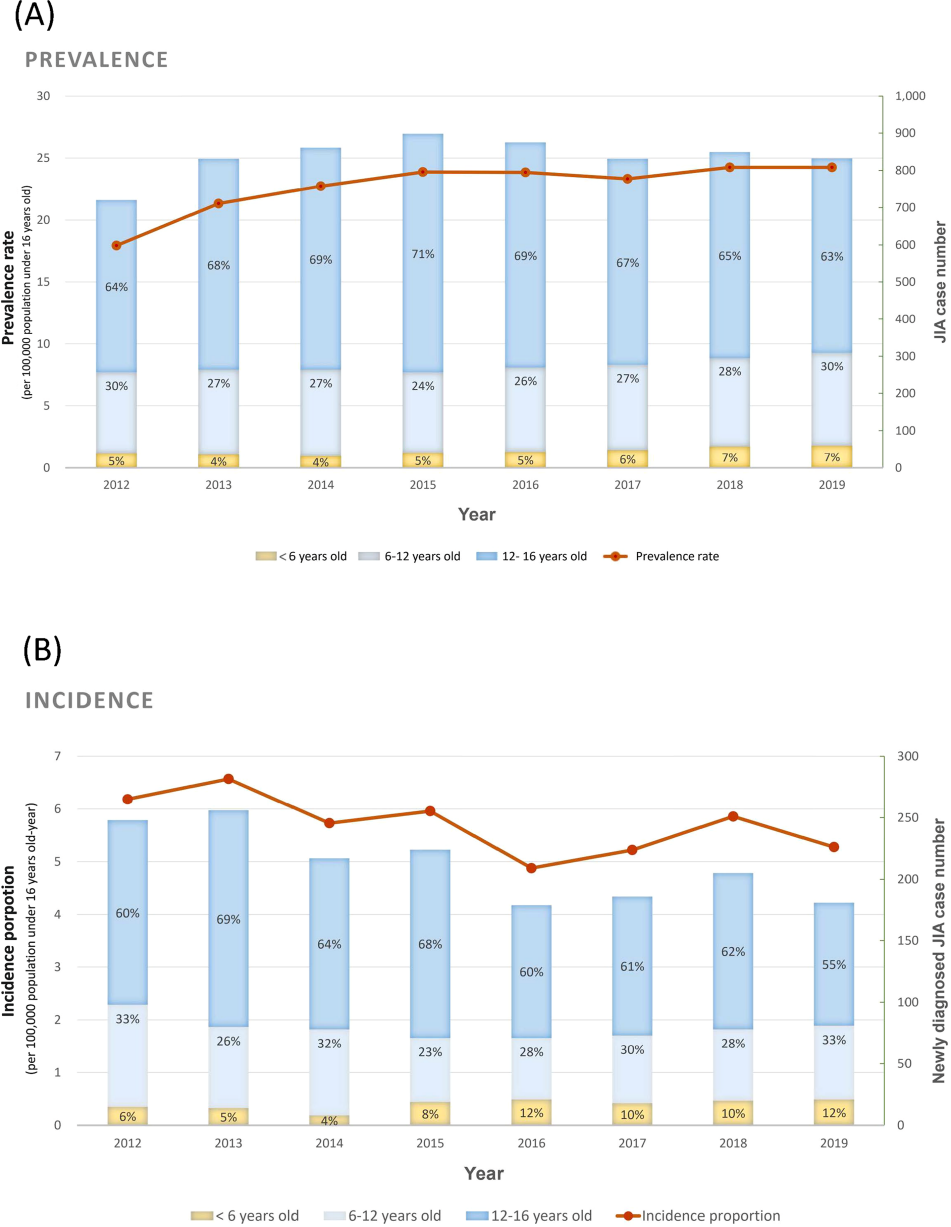
Prevalence **(A)** and incidence **(B)** of juvenile idiopathic arthritis (JIA) in Taiwan from 2012 to 2019. Bar plots display the ratios of three age categories at onset: under 6 years, 6 to 12 years, and 12 to 16 years.

During the review period from 2012 to 2019, 306,254 patients who met the criteria had enrollment records in the registry for beneficiaries. A total of 2,033 JIA patients were identified from the NHIRD over the 8-year study period, and 1,696 patients were newly diagnosed with JIA after January 1st, 2012 (**Supplementary Figure S1**). The JIA prevalence rate ranged from 18.0 to 24.2 per 100,000 population ≤16 years old (**Figure 1A**), with a male predominance (male to female ratio: 1.08-1.52:1). The annual incidence proportion ranged from 5.22 to 6.57 per 100,000 population ≤16 years old-year with an overall incidence rate of 4.54 per 100,000 person-year from 2012 to 2019 (**Figure 1B**). The trend of incidence rate and total fresh JIA patient numbers gradually decreased year by year. More than half of the patients had their diagnosis after 12 years old. However, an increase in the proportion of patients diagnosed under six years old was observed.

The baseline characteristics of the patients in the JIA incident cohort are summarized in **Table 1**. Our cohort revealed a median diagnostic age of 13, ranging from 1 to 15 years old. Two-thirds of patients were 12–16 years old at diagnosis. The patients with JIA had a higher proportion of males. We observed no particular difference in diagnostic case numbers between months and seasons. The geographic distribution at diagnosis showed a cluster in northern Taiwan (53.71%) with a predominance in Urban areas.

**Table 1:** Demographic and clinical characteristics of JIA patients stratified by subgroups (JIA-SpA vs. Non-SpA).

Characteristics		Incident cohort			
		Total population N=*1,696*	JIA-SpA	Non-SpA	*p-value*
			N=835	N=861	
Age at diagnosis, years old	Median (IQR)	13.0 (3.0)			
Age groups at diagnosis, years old	< 6	115 (6.8%)	7 (0.8%)	108 (12.5%)	<0.0001
	6 to <12	429 (25.3%)	122 (14.6%)	307 (35.7%)	
	12 to ≤16	1,152 (67.9%)	706 (84.6%)	446 (51.8%)	
Sex	Male	967 (57.0%)	586 (70.7%)	381 (44.4%)	<0.0001
	Female	721 (42.5%)	243 (29.3%)	478 (55.6%)	
	Missing	8 (0.5%)	6	2	
Comorbidities	Uveitis	87 (5.1%)	61 (7.3%)	26 (3.0%)	<0.0001
	Psoriasis	24 (1.4%)	24 (2.9%)	0 (%)	NA
Medical specialty visited at diagnosis	Pediatrics	743 (43.8%)	185 (22.2%)	558 (64.8%)	<0.0001
	Rheumatology	537 (31.7%)	390 (46.7%)	147 (17.1%)	
	Orthopaedics	271 (16.0%)	165 (19.8%)	106 (12.3%)	
	Physical Medicine and Rehabilitation	46 (2.7%)	37 (4.4%)	9 (1.0%)	
	Internal medicine	33 (2.0%)	25 (3.0%)	8 (0.9%)	
	Dermatology	8 (0.5%)	7 (0.8%)	1 (0.1%)	
	Others	58 (3.4%)	26 (3.1%)	32 (3.7%)	
Levels of hospital visited at diagnosis	Medical center	979 (57.7%)	400 (47.9%)	579 (67.2%)	<0.0001
	Regional hospital	412 (24.3%)	253 (30.3%)	159 (18.5%)	
	District Hospital	159 (9.4%)	104 (12.5%)	55 (6.4%)	
	Local clinic	141 (8.3%)	74 (8.9%)	67 (7.8%)	
	Missing	5 (0.3%)	4	1	

For quantitative data, median and IQR are presented.

### Demographic and clinical differences between subgroups

Using the operational definition, we identified 835 patients as the JIA-SpA subgroup, representing 49.2% of the incident cohort. The JIA-SpA and Non-SpA subgroups showed distinct demographic and healthcare utilization profiles (**Table 1**). The JIA-SpA subgroup had significant male predominance (70.7% vs. 44.4%) and late disease onset, with most patients diagnosed between ages 12 and 16 (*p* < 0.0001). In contrast, the Non-SpA group included more females and younger children. Regarding extra-articular manifestations, the JIA-SpA subgroup had a significantly higher prevalence of uveitis than the Non-SpA subgroup (7.3% vs. 3.0%, *p* < 0.0001). The odds of developing uveitis were approximately 2.5 times higher in the JIA-SpA group.

Significant disparities emerged in the site of diagnosis (*p* < 0.0001). Non-SpA patients were predominantly managed in pediatric departments (64.8%) at medical centers (67.2%). However, JIA-SpA patients frequently sought care in adult rheumatology (46.7%) or orthopedics (19.8%) departments. JIA-SpA patients were also more likely to be diagnosed at regional or district hospitals (combined 42.8%) than the Non-SpA group (24.9%), indicating a more decentralized care pattern.

### JIA treatment distribution

We analyzed JIA medications used for more than three consecutive months in the prevalent cohort as the chronic treatments for JIA (**Supplementary Table 3**). Among 2,033 patients, 1,255 (61.73%) patients had continuously used at least one JIA medication for over three months. The most frequently prescribed medication was NSAIDs (51.11%), followed by methotrexate (MTX) (32.22%) and sulfasalazine (26.96%). Systemic glucocorticoids, less suggestive as a long-term treatment for JIA, were used in 24.05% of cases. TNFα inhibitors were the most commonly used biologics, with a similar proportion in etanercept and adalimumab prescriptions, at 11.71% and 10.23%, respectively.

Chronic medication usage revealed distinct treatment patterns between the two subgroups (**Supplementary Table 3**). Among csDMARDs, Sulfasalazine was the predominant agent in the JIA-SpA subgroup, used by 38.8% of patients, compared to 16.0% in the Non-SpA subgroup (*p* < 0.001). Conversely, Methotrexate was more prevalent in the Non-SpA subgroup (38.9% vs. 25.0%, *p* < 0.001). The Non-SpA subgroup also had a higher rate of systemic glucocorticoid use (27.8% vs. 20.0%, *p* < 0.001), likely reflecting the inclusion of systemic and polyarticular phenotypes that require more intensive anti-inflammatory control. Among biologic therapies, while TNF inhibitors were used in both groups, Tocilizumab was predominantly prescribed in the Non-SpA subgroup (4.5% vs. 1.2%, *p* < 0.001), consistent with its indication for systemic JIA.

### Biologic treatment patterns: overall and subgroup comparison

**Figure 2:**
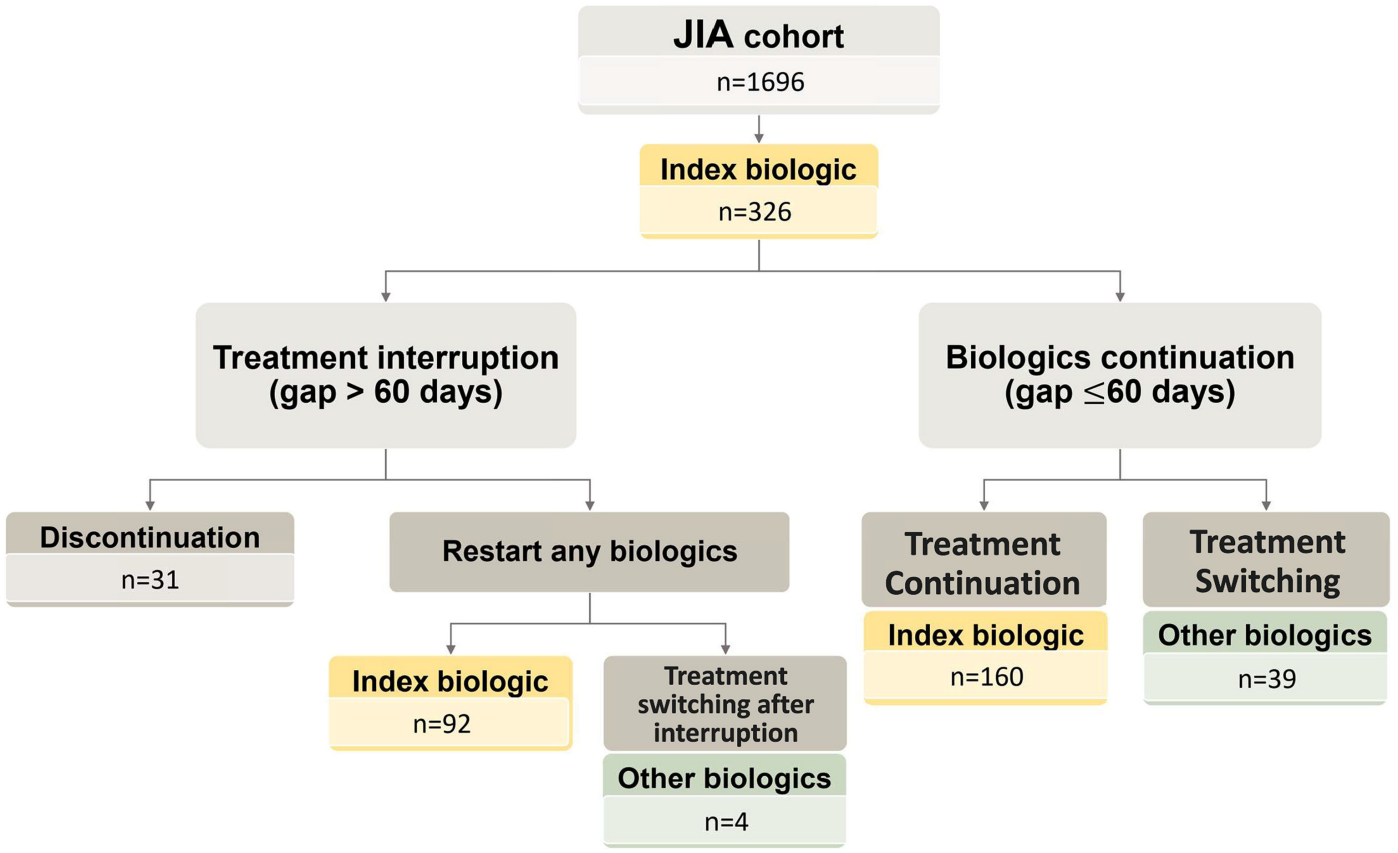
Flow chart of biologics use in patients with juvenile idiopathic arthritis (JIA) and the treatment course of the 326 JIA patients receiving biologics. The data are expressed as patient numbers.

In the incident cohort containing 1,696 cases, 326 (19.2%) patients received documented biologics, either etanercept, adalimumab, or tocilizumab. **Figure 2** shows the flowchart of biologic treatment patterns. **Supplementary Figure S2** shows the KM curve for biologics use after diagnosis. The median of the 1-year, 3-year, and 5-year medication rates for any biologics were 11.7%, 17.0%, and 19.3%. **Table 2** lists the treatment patterns for the three biologics. The median time (IQR) from diagnosis to first biologics use was 0.73 (1.22), 0.71 (1.24), 0.80 (1.19), and 0.66 (0.92) years for all biologics, etanercept, adalimumab, and tocilizumab, respectively. The median duration of biologics use ranged from 1.49 to 2.24 years, with the longest duration in the etanercept-treated group. A combination of csDMARDs was common in the first year of biologics treatment, with the combination rate of csDMARDs decreasing to about half of the patients after the first year of treatment. While the patients treated with tocilizumab had the highest combination rate, the differences between the three biologics were insignificant.

**Table 2:** The treatment pattern of the first prescribed biologic in the JIA incident cohort.

Variables		Incident cohort N = 1,696		
		Etanercept	Adalimumab	Tocilizumab
Patient number		136	166	24
Time from diagnosis to index biologic use, year		0.71 (1.24)	0.80 (1.19)	0.66 (0.92)
Duration of the index biologic use, year		2.24 (2.42)	1.49 (2.25)	1.80 (2.12)
Treatment combined csDMARDs	In the first year	124 (91.18%)	148 (89.16%)	≥21 ^a^ (N/A)
	After the first year	67 (49.26%)	84 (50.60%)	14 (58.33%)
Treatment continuation, switching, restart, and discontinuation				
Treatment continuation		51 (37.5%)	99 (59.6%)	≥8 ^a^ (N/A)
Treatment switching		24 (17.6%)	14 (8.4%)	≤3 ^a^ (N/A)
Restart any biologics after interruption		48 (35.3%)	39 (23.5%)	9 (37.5%)
Discontinuation		13 (9.6%)	14 (8.4%)	4 (16.7%)
Discontinuation rate (95% CI)	1-year	17.2% (11.4- 24.1%)	22.0% (15.8-28.9%)	25.0% (9.9-43.6%)
	3-year	52.6% (43.2-61.2%)	44.2% (35.1-53.0%)	61.3% (35.4-79.4%)
	5-year	74.3% (63.5-82.3%)	61.0% (47.5-72.0%)	74.2% (32.4-92.4%)

The data presented index biologics use only. For quantitative data, median and IQR are presented.

a All non-zero counts that were less than three were suppressed to protect patient privacy.

CI, confidence interval; N/A, not applicable; csDMARDs, conventional synthetic disease-modifying anti-rheumatic drugs.

Due to the unavailability of individual patient-level data for log-rank analysis, we conducted a comparative analysis with aggregated data. We found that a higher proportion of patients in the Non-SpA subgroup received biologics than in the JIA-SpA subgroup (23.1% vs. 15.2%, *p* < 0.001). Adalimumab was the most commonly used in both groups (~50%), while tocilizumab use was lower in the JIA-SpA subgroup (3.9% vs. 9.5%), reflecting guidelines for axial involvement, though this difference was not statistically significant (*p* = 0.08). For treatment retention, patients with JIA-SpA on adalimumab had a higher continuation rate than Non-SpA patients (65.2% vs. 56.0%, *p* = 0.31), suggesting better long-term drug survival for the SpA phenotype, though the limited sample size affected significance.

### Drug survival of JIA biologics

**Table 2** shows the proportions of biologics continuation, switching, restart, and discontinuation. We compared the distribution of treatment courses for the two TNFα inhibitors and identified significant differences between the etanercept and the adalimumab groups (*p*-value=0.001). Patients using adalimumab showed a higher continuous treatment course, whereas a greater number of patients using etanercept experienced biologic switching and restart.

**Figure 3:**
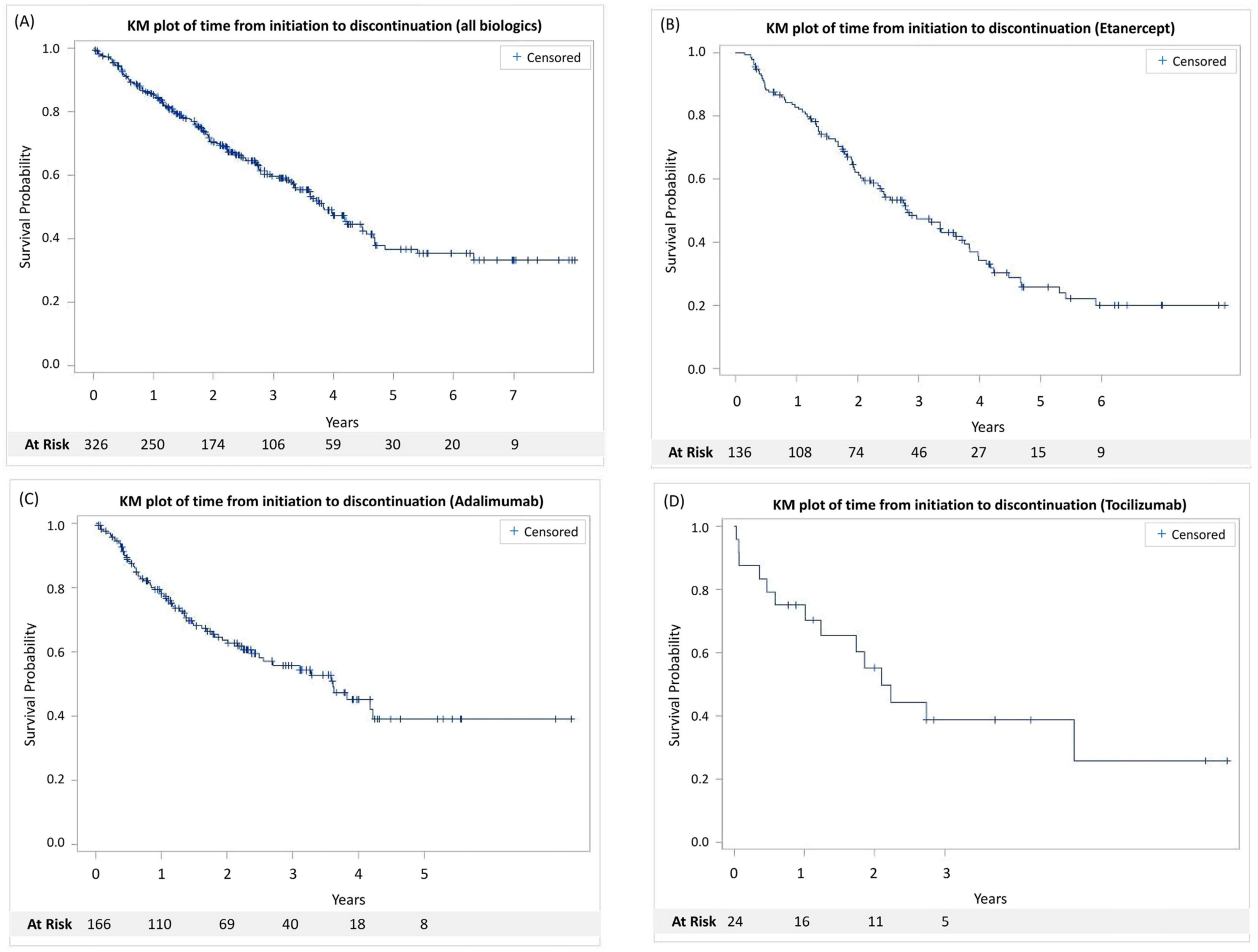
Kaplan-Meier curves illustrating drug survival for biologics utilized in juvenile idiopathic arthritis patients. Curves are presented for **(A)** all biologics, **(B)** etanercept, **(C)** adalimumab, and **(D)** tocilizumab.

Among 326 patients using biologics, 199 (61.0%) patients used biologics without interruption. The overall biologics continuation rate was 49.08%. A summary of the cumulative 1-, 3-, and 5-year discontinuation rates for 326 treatment courses is presented in **Table 2**. Etanercept had the lowest 1-year discontinuation rate of 17.2% (95% CI, 11.4-24.1%). However, adalimumab had the lowest 3-year discontinuation rate of 44.2% (95% CI, 35.1-53.0%), which consistently sustained to the fifth year at 61.0% (95% CI, 47.5-72.0%). **Figure 3** shows the KM analysis demonstrating crude drug discontinuation of each biologic and all biologics. Patients discontinued their index biologics, or any biologics were defined as an event. The median discontinuation time was 2.81 years, 3.61 years, and 2.10 years in the etanercept, adalimumab, and tocilizumab groups. Considering all biologics, the median discontinuation time was 3.83 years.

During the follow-up period, 43 patients had a switch of biologics. Thirty-nine patients (90.6%) switched their treatment within the 60-day gap, and 32 of the 39 patients changed their index biologics within a 14-day gap after using them. In 24 etanercept-treated patients with treatment switch, 17 switched to adalimumab, while fewer switched to tocilizumab (7 patients). Treatment switching was less common in the adalimumab group; 10 out of 14 patients switched to tocilizumab, and the rest switched to etanercept (≤3 patients) or abatacept (≤3 patients). In the tocilizumab group, only a few patients switched to other JIA biologics (≤3 patients). **Supplementary Figure S3** shows the KM plot of time from drug initiation to switch treatment in each biologic.

The restart of biologics was observed in 96 patients. Most patients reused the same drugs before an interruption of over 60 days, with a ratio of more than 90% in the etanercept and adalimumab groups, and 100% in the tocilizumab group. **Supplementary Figure S4** shows the KM plot of time from drug interruption to restart in all biologics, and the median restart time was 0.16 years.

## Discussion

Epidemiological data for JIA reveal notable variation across geographic regions and different JIA subtypes. Thierry et al. reported an average annual incidence rate of 7.8 patients per 100,000 person-years (95% CI, 7.6-8.1%) from the ACR, ILAR, and EULAR datasets between 1983 and 2010 (3). In Taiwan, national surveys indicated an incidence of 4.39 per 100,000 person-years (range: 3.93-6.23) from 1999 to 2009 person-years (14), with a prevalence of 3.8 per 100,000 population (95% CI: 3.3-4.3%) in 2009 (15). Our study found a higher incidence and prevalence of JIA from 2011 to 2019, although these figures were still lower than those observed in the western countries. A male predominance was also observed, consistent with findings from other research conducted in Taiwan (4, 5, 14), which contrasts with the trend of higher female patients typically seen in Western studies (3, 16). This discrepancy may be linked to the prevalence of ERA in Taiwan (4, 5). In addition, while the onset age for JIA globally is around 10 years (16, 17), our study findings indicate an onset age of 13 years. This aligns with the later onset typically associated with ERA compared to other JIA subtypes. Overall, our study findings elucidate a distinct ERA-dominant distribution pattern of JIA in Taiwan, emphasizing the need for tailored diagnostic and treatment strategies in the region.

The analysis of drug distribution data indicates a treatment pattern consistent with the ACR guidelines for JIA. These guidelines recommend MTX over other DMARDs for escalating therapy in all JIA subtypes except systemic JIA (7, 8). The percentage of MTX use in our cohort (32.2%) is lower than that reported in other international JIA registries, which range from 61.0% to 84.2% (17). Shih et al. reported that 72% of JIA patients at a single Taiwanese medical center used MTX from 1993 to 2018 (5). Our analysis focused only on chronic treatments of JIA lasting longer than three months, leading to an underestimation of MTX usage. Since the NHIRD lacks detailed information on disease severity and the adverse effects of JIA medications, further investigation into the underutilization of MTX is warranted. In our findings, sulfasalazine was the second most commonly used DMARD (27.0%) and predominant in the JIA-SpA subgroup (38.8%). The medication profiles in our study reflect real-world adherence to treatment guidelines. The preference for sulfasalazine over methotrexate in the JIA-SpA subgroup aligns with evidence supporting sulfasalazine’s efficacy in peripheral spondyloarthritis and enthesitis-related arthritis (16, 18). Although sulfasalazine was not required for reimbursement application, nearly 27% of patients in Taiwan were using it due to the high prevalence of ERA and its greater effectiveness in that specific subtype.

Our research found that 19.2% of JIA patients had received biologics for at least three months. A retrospective study encompassing 722 JIA patients from a multi-institutional database in Taiwan yielded comparable results, with 16.8% of patients undergoing biologics treatment between 2001 and 2019 (19). This prevalence of biologics use is lower than the figures reported in recent cohort studies in North America and Europe during the biologic era, where the range was between 24.0% and 61.8% of JIA patients prescribed at least one biologic (16, 17). The variability in the utilization of biologics may be attributed to the reimbursement policies governing the use of JIA biologics in Taiwan. Clinically, patients are typically required to undergo at least three months of prior medication to qualify for biologic reimbursement. Subsequently, those who do not achieve adequate response to MTX may initiate the reimbursement application process, contingent on its approval. Given the considerable cost associated with biologics as a long-term treatment for JIA, only a limited number of patients in Taiwan can afford to self-finance their biologic treatments. Beyond reimbursement policies, the specific distribution of JIA subtypes in Taiwan also contributes to the observed treatment patterns. Our subgroup analysis revealed a significantly lower biologic utilization rate in the JIA-SpA subgroup (15.2%) compared to the Non-SpA subgroup (23.1%). Adolescents with ERA often present with enthesitis or inflammatory back pain—manifestations that do not qualify as entry criteria for biologic reimbursement. Given the greater disease severity and less favorable long-term outcomes of ERA compared to most other JIA subtypes (20, 21), this disparity likely reflects unmet treatment needs among Taiwanese ERA patients requiring biologic therapy.

Most countries predominantly use a “step-up” approach in the management of JIA. However, some clinical studies have demonstrated the significant advantages of early initiation of biologics when compared to the standard step-up regimen in achieving disease remission (22–24), particularly in high-risk patient groups such as those diagnosed with polyarthritis and systemic JIA (25). This highlights the necessity for timely biologic intervention based on the disease trajectory and treatment response to improve long-term patient outcomes. Furthermore, the National Health Insurance does not cover several biologics for JIA treatment, such as anakinra, canakinumab, golimumab, rituximab, or secukinumab. As a result, there is a lack of understanding regarding the application of these biologics within the Taiwanese population. It is also notable that patients with ERA exhibit more persistent disease manifestations, higher pain scores, and poorer health status than the other JIA subtypes (5, 20, 26). Therefore, the development of therapeutic agents and the refinement of treatment strategies, particularly for ERA, are imperative for enhancing clinical outcomes.

In our cohort, biologics switching was observed in 13% of JIA patients using biologics, which is lower than the rates reported by the Childhood Arthritis and Rheumatology Research Alliance Registry (CARRA) (26%) (27) and the national survey in the UK (23%) (28), where etanercept was the predominant initial biologic (29). Our study involved a more recent cohort of JIA patients, with adalimumab as the most commonly prescribed biologic, reflecting a lower switching rate compared to etanercept. This trend of lower switching rates was also observed in the CARRA and UK registry studies (27, 28). The main reasons for switching treatment include ineffective response, disease flares, and adverse effects. The most common reason for switching biologics was ineffectiveness or a disease flare observed in 58-60% of the patients (27, 28). The transitions from etanercept and adalimumab to tocilizumab were likely due to TNFα inhibitor failure, leading in a switch to another mechanism of action. Nevertheless, the conclusions drawn from our findings are constrained by the limited sample size and the absence of detailed clinical information.

Our study draws strength from its substantial sample size and comprehensive coverage of the NHIRD, facilitating a population-based analysis of JIA treatment patterns. The longitudinal NHIRD data afford valuable insights into long-term management and healthcare policy decisions for JIA in the Taiwanese population. The study has certain limitations. First, misclassification bias can be a concern due to potential inaccuracies in diagnosis coding, and issues related to data quality may lead to inaccuracies in the recorded information. To mitigate the issue of inaccurate diagnoses, we have established stringent inclusion criteria for identifying JIA patients. These criteria integrate multiple clinic visits and the use of NSAIDs, which are commonly used in the initial treatment of JIA, to ensure a more reliable diagnosis. Furthermore, the databases lack essential clinical information, such as lab results, JIA subtypes, and severity. It may also fail to provide accurate insights into patients’ treatment adherence. Another important limitation is that we relied on ICD codes for subgroup classification. The ILAR classification criteria for ERA and psoriatic arthritis require detailed clinical information that is unavailable in NHIRD data. However, our findings strongly validate the operational definition of the JIA-SpA subgroup. Despite the lack of serological data (e.g., HLA-B27) in the NHIRD, the identified JIA-SpA cohort exhibited a striking male predominance (70.7%) and adolescent onset—both hallmark features of ERA and juvenile SpA reported in the literature (5, 30). Additionally, the exclusive presence of psoriasis in the JIA-SpA subgroup (2.9%) validates our classification algorithm and ensures that the Non-SpA subgroup remains a relatively homogeneous cohort of oligoarticular, polyarticular of systemic JIA without psoriatic or enthesitis-related features.

It is also important to note that medication selection in Taiwan is driven by policy, which may limit the external validity of our findings. Taiwan’s biologic reimbursement policies align with current treatment recommendations for non-systemic JIA from ACR, EULAR, and Japanese guidelines. These guidelines recommend MTX as the preferred initial csDMARD globally, with biologics added for inadequate response after 3–6 months of treatment (7, 8). However, Taiwan’s National Health Insurance review process may result in lower biologic uptake among JIA patients with persistent symptoms but normal inflammatory markers or minimal arthritis signs. Additionally, out-of-pocket medications are not captured in the database, which further limits the comprehensiveness of the data. Future research should address these limitations by integrating more detailed clinical data through linkage with electronic health records or prospective cohort studies, enabling more nuanced and efficacious management strategies for JIA patients.

## Conclusions

In summary, the analysis of JIA from a national cohort over the 8-year study period reveals significant epidemiological insights and treatment patterns. These insights underscore the necessity for region-specific and personalized management strategies tailored to the unique epidemiological characteristics of JIA in Taiwan. Integrating more detailed clinical data in future research can enhance understanding and improve treatment outcomes for JIA patients, ultimately contributing to better healthcare policies and practices in managing this chronic condition.

## Data Availability

The data analyzed in this study is subject to the following licenses/restrictions: This study was obtained from the National Health Insurance Research Database (NHIRD) of Taiwan. Because of regulations designed to protect patient privacy, the database has restrictions and is not publicly available. We provided the codes for statistical analysis on the Github: https://github.com/yun-lin-h/JIA. Requests to access these datasets should be directed to https://dep.mohw.gov.tw/DOS/cp-2506-3633-113.html.
