# Supplementary material for: The epidemiology and biologics treatment patterns of juvenile idiopathic arthritis in Taiwan- an 8-year follow-up
Source: Front Immunol. 2025 Dec 16;16:1712103. doi: 10.3389/fimmu.2025.1712103 (PMC12747926; doi:10.3389/fimmu.2025.1712103)
Supplement: Supplementary file 1 [file Supplementaryfile1.docx]

**Supplementary data**

The codes for statistical analysis are provided on the Github: https://github.com/yun-lin-h/JIA

Supplementary Table 1. **The ICD codes used for the classification criteria of the JIA patient population and the JIA-SpA subgroup.**

|  | **ICD-9 code** | **ICD-10 code** |
| --- | --- | --- |
| **JIA** | 714.0: Rheumatoid arthritis  714.2: Other rheumatoid arthritis with visceral or systemic involvement  714.3: Juvenile chronic polyarthritis  720.0: Ankylosing spondylitis  720.9: Unspecified inflammatory spondylopathy  720.89: Other inflammatory spondylopathies  720.2: Sacroiliitis, not elsewhere classified  696.0: Psoriatic arthropathy | M05: Rheumatoid arthritis with rheumatoid factor  M06: Other rheumatoid arthritis  M08: Juvenile arthritis  M45: Ankylosing spondylitis  M46.1: Sacroiliitis, not elsewhere classified  M46.8: Other specified inflammatory spondylopathies  M46.9: Unspecified inflammatory spondylopathy  L40.50: Arthropathic psoriasis, unspecified |
| **JIA-SpA** | 720.0: Ankylosing spondylitis  720.9: Unspecified inflammatory spondylopathy  720.89: Other inflammatory spondylopathies  720.2: Sacroiliitis, not elsewhere classified  696.0: Psoriasis and similar disorders | M08.1: Juvenile ankylosing spondylitis  M45: Ankylosing spondylitis  M46.1: Sacroiliitis, not elsewhere classified  M46.8: Other specified inflammatory spondylopathies  M46.9: Unspecified inflammatory spondylopathy  L40.50: Arthropathic psoriasis, unspecified |

Supplementary Table 2. **The treatments for JIA and related ATC codes**

| Drug name | ATC codes |
| --- | --- |
| NSAIDs | M01A |
| Steroids | H02, M01BA |
| csDMARDs |  |
| Sulfasalazine | A07EC01 |
| Methotrexate | L04AX03 |
| Mycophenolate mofetil / Mycophenolic acid | L04AA06 |
| Cyclosporine | L04AD01 |
| Hydroxychloroquine | P01BA02 |
| Azathioprine | L04AX01 |
| Biologics |  |
| Etanercept | L04AB01 |
| Adalimumab | L04AB04 |
| Tocilizumab | L04AC07 |
| Abatacept | L04AA24 |

Supplementary Table 3**. JIA medications used for over three months during the follow-up periods**

|  | Total population *N=2,033* | JIA-SpA *N*=976 | Non-SpA *N*=1057 | *p-*value |
| --- | --- | --- | --- | --- |
| NSAIDs | 1,039 (51.1%) | 535 (54.8%) | 504 (47.7%) | 0.001 |
| Systemic glucocorticoids | 489 (24.1%) | 195 (20.0%) | 294 (27.8%) | < 0.001 |
| csDMARDs |  |  |  |  |
| Methotrexate | 655 (32.2%) | 244 (25.0%) | 411 (38.9%) | < 0.001 |
| Sulfasalazine | 548 (27.0%) | 379 (38.8%) | 169 (16.0%) | < 0.001 |
| Hydroxychloroquine | 220 (10.8%) | 62 (6.4%) | 158 (14.9%) | < 0.001 |
| Azathioprine | 101 (5.0%) | 20 (2.0%) | 81 (7.7%) | < 0.001 |
| Cyclosporine | 28 (1.4%) | 11 (1.1%) | 17 (1.6%) | 0.35 |
| Mycophenolate mofetil/ Mycophenolic acid | 6 (0.3%) | 0 (0.0%) | 6 (0.6%) | 0.03 |
| Biologics |  |  |  |  |
| Etanercept | 238 (11.7%) | 85 (8.7%) | 153 (14.5%) | < 0.001 |
| Adalimumab | 208 (10.2%) | 82 (8.4%) | 126 (11.9%) | 0.011 |
| Tocilizumab | 60 (3.0%) | 12 (1.2%) | 48 (4.5%) | < 0.001 |
| Abatacept | 9 (0.4%) | ≤3 | ≥6 | N/A |

Medication supplied for more than 3 consecutive months of each drug was included in the analysis. NSAIDs, non-steroidal anti-inflammatory drugs; csDMARDs, conventional synthetic disease-modifying anti-rheumatic drugs.
^a^ All non-zero counts that were less than three were suppressed to protect patient privacy.

**Supplementary Table 4. Comparison of biologics use between JIA-SpA and Non-SpA subgroups in the incident cohort.**

|  | | Incident cohort | | |
| --- | --- | --- | --- | --- |
|  | | JIA-SpA  (N=835) | Non-SpA (N=861) | *p-value* |
| Biologics use, n (%) | | 127 (15.2%) | 199 (23.1%) | <0.001 |
| Index biologics | Etanercept, n (%) | 56 (44.1%) | 80 (40.2%) | 0.49 |
|  | Adalimumab, n (%) | 66 (52.0%) | 100 (50.3%) | 0.76 |
|  | Tocilizumab, n (%) | 5 (3.9%) | 19 (9.5%) | 0.08^#^ |

# analyzed by Fisher's Exact Test

**Supplementary Table 5. The treatment pattern of the first prescribed biologic in the JIA-SpA incident cohort.**

|  | | Incident cohort N=835 | | |
| --- | --- | --- | --- | --- |
|  | | Etanercept | Adalimumab | Tocilizumab |
| Patient number | | 56 | 66 | 5 |
| Time from diagnosis to index biologic use, year | | 1.28 (2.44) | 1.17 (2.10) | 1.73 (0.59) |
| Duration of the index biologic use, year | | 2.56 (2.21) | 1.72 (2.30) | 1.01 (0.78) |
| Treatment combined csDMARDs | In the first year | 54 (96.43%) | 54 (81.82%) | 5 (100%) |
|  | After the first year | 35 (62.50%) | 32 (48.48%) | ≤3 ^a^ (N/A) |
| Treatment continuation, switching, restart, and discontinuation | | | | |
| Treatment continuation | | 22 (39.2%) | 43 (65.2%) | 0 (0%) |
| Discontinuation rate (95% CI) | 1-year | 11.1%  (4.5- 21.2%) | 16.6%  (8.5-27.1%) | 40% (3.1-78.6%) |
|  | 3-year | 50.0%  (35.0-63.4%) | 43.2%  (28.6-57.0%) | 0%  (N/A) |
|  | 5-year | 75.0%  (55.6-86.8%) | 47.5%  (31.2-62.2%) | 0%  (N/A) |

The data presented index biologics use only. For quantitative data, median and IQR are presented.
CI, confidence interval; N/A, not applicable; csDMARDs, conventional synthetic disease-modifying anti-rheumatic drugs.

**Figure legends:**

Supplementary Figure 1. The algorithm for enrollment and inclusion of the JIA and JIA-SpA study population.

Supplementary Figure 2. Kaplan-Meier survival graph of JIA patients from diagnosis to use of the index biologics.

Supplementary Figure 3. Kaplan-Meier survival graph of JIA patients from initiating the index biologics to switching to other biologics. The graph includes patients with (A) all biologics, (B) etanercept, (C) adalimumab, and (D) tocilizumab.

Supplementary Figure 4. Kaplan-Meier survival graph of JIA patients with any biologics from the time of biologics interruption to biologics restart.

Figure S1


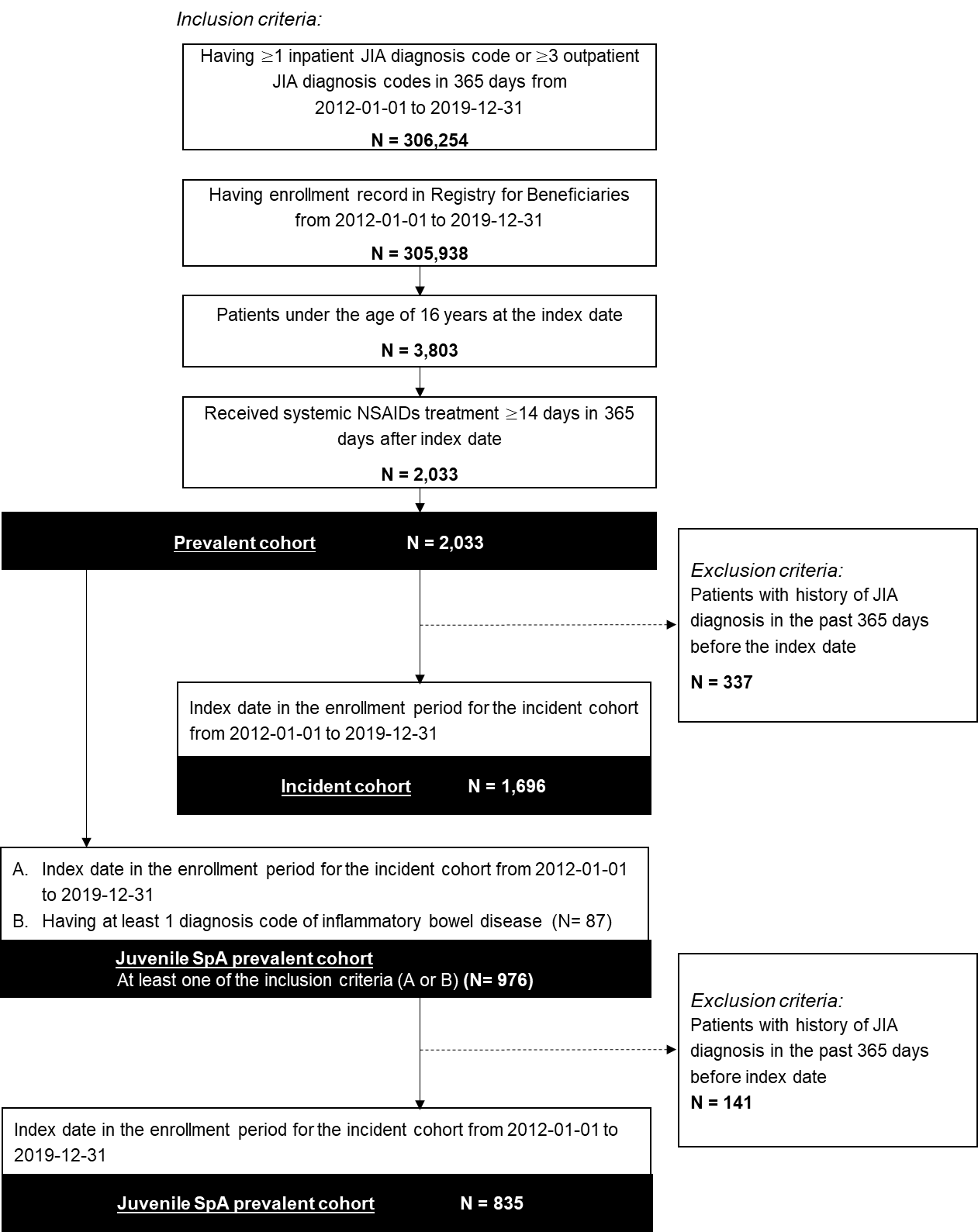


Figure S2


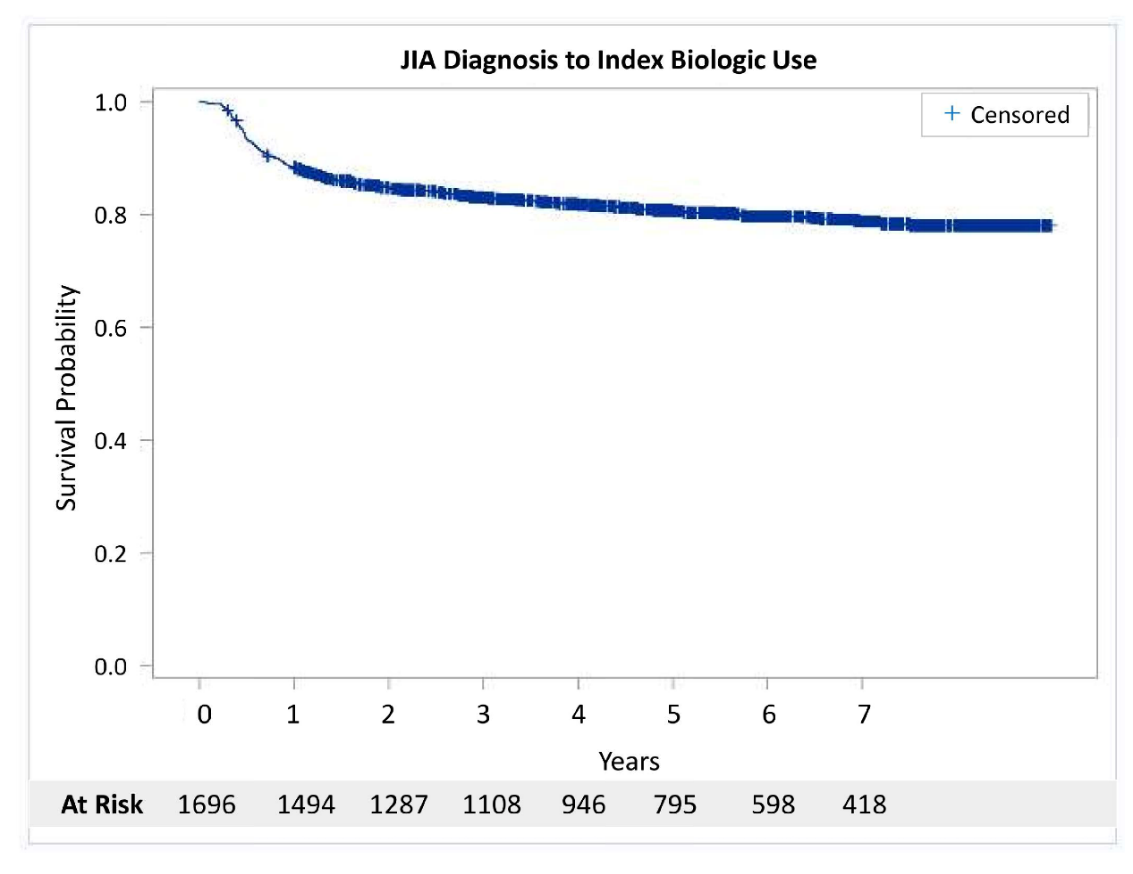


Figure S3


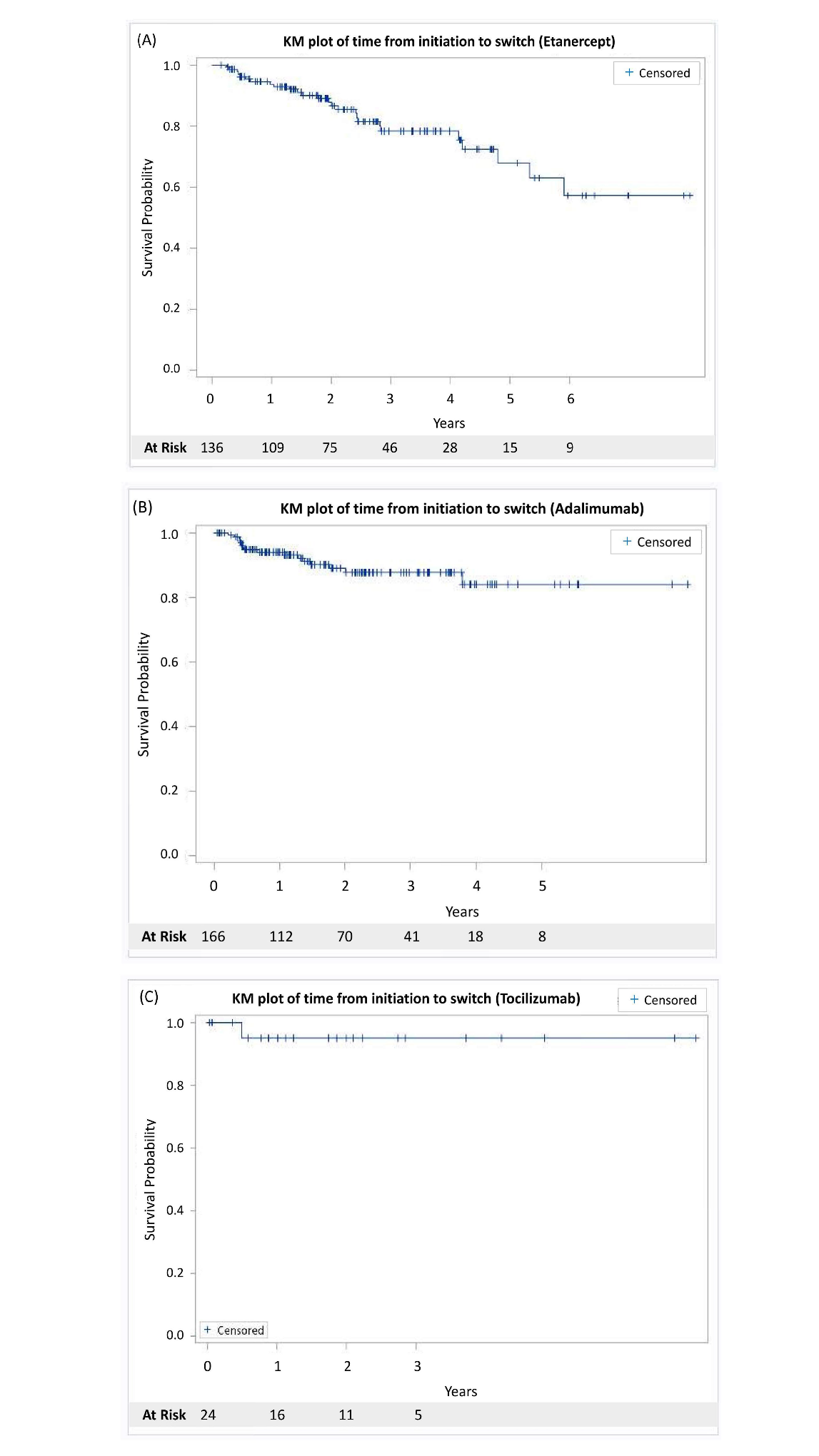


Figure S4


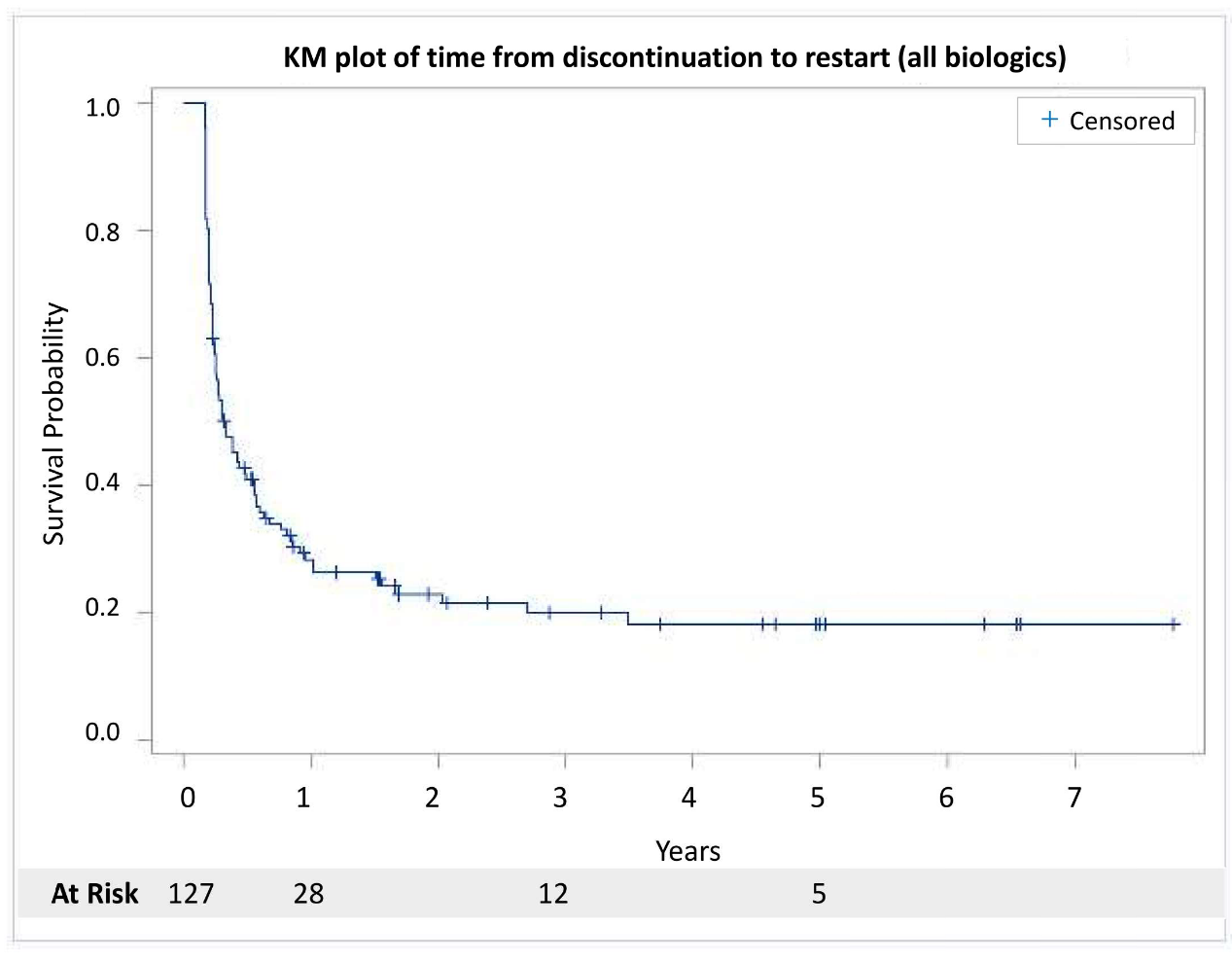

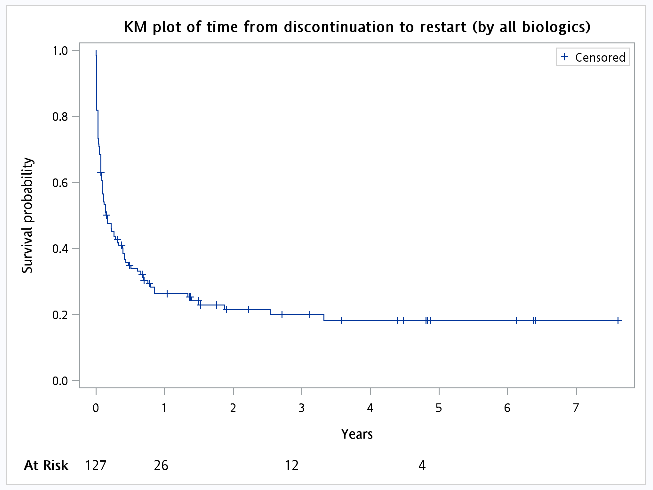


**At Risk** 127 26 12 4
